# Molecular damage in cancer: an argument for mTOR-driven aging

**DOI:** 10.18632/aging.100422

**Published:** 2011-12-31

**Authors:** Mikhail V. Blagosklonny

**Affiliations:** Department of Cell Stress Biology, Roswell Park Cancer Institute, Elm and Carlton Streets, Buffalo, NY, 14263, USA

**Keywords:** cancer, target, therapy, leukemia, anticancer drugs

## Abstract

Despite common belief, accumulation of molecular damage does not play a key role in aging. Still, cancer (an age-related disease) is initiated by molecular damage. Cancer and aging share a lot in common including the activation of the TOR pathway. But the role of molecular damage distinguishes cancer and aging. Furthermore, an analysis of the role of both damage and aging in cancer argues against “a decline, caused by accumulation of molecular damage” as a cause of aging. I also discuss how random molecular damage, via rounds of multiplication and selection, brings about non-random hallmarks of cancer.

## INTRODUCTION

Aging is defined as a decline caused by accumulation of all sorts of damage, in particular, molecular damage. This statement seemed so obvious that it was not questioned. Yet several lines of evidence rule out molecular damage as a cause of aging [1-15]. Yes, of course, molecular damage accumulates over time. But this accumulation is not sufficient to cause organismal death. Eventually it would. But the organism does not live long enough, because another cause terminates life first [8]. This cause is aging, a continuation of developmental growth. Definitely, developmental growth is not driven by accumulation of molecular damage, although molecular damage accumulates. Similarly, aging is not driven by damage.

Growth is stimulated in part by mitogen- and nutrient-sensing (and other) signaling pathways such as mTOR [16-35]. Aging, “an aimless continuation of developmental program”, is driven by the same signaling pathways including mTOR [8, 14, 24]. Aging in turn causes damage: not molecular damage but non-random organ damage (stroke, infarction, renal failure and so on) and death [13]. Seemingly, one objection to this concept is that cancer is caused by molecular damage. And cancer is often a cause of death in mammals. So how may one claim that damage does not drive aging, if it is involved in cancer. Let us discuss this.

### Damage in cancer

Damage causes activate oncogenes and de-activate tumor suppressors due to genetic mutations, epigenetic alterations and microRNAs dysregulation [36-57]. Even according to alternative theories, cancer is caused by damage too [58]. So damage is involved in cancer. There are some exceptions, mostly related to embryonic cells. Also, in theory, extra-genetic alterations such as stable activation of oncogenic pathways via positive feedback loops can contribute to malignant phenotype [59]. Finally, positive feedback loops could be established between cancer and normal cells [59-61]. But in general molecular damage is a key factor in cancer origin. In agreement, cancer is associated with genetic instability [59, 62-69].

### Not decline but robustness

Due to genetic instability, cancer cells accumulate high levels of unrepaired damage, resulting in genomic mutations and epigenetic alterations as well as aneuploidy [36-49, 70-80]. Despite of accumulation of damage, cancer is neither decline nor ‘wear and tear’. Cancer cells are robust and aggressive. Cancer cells damage organs, thus killing organism. If cancer cells with all damage are so robust, then how possibly aging of normal cells could be “a decline due to accumulation of molecular damage”. In fact, it does not.

### Immortality of cancer cells

Cancer is associated with cellular immortality [38, 81-88]. Not only cancer cells can become cell lines but also they can become free-living organisms [89-96]. Such free-living cancer cells spread from one animal to another. Thus, venereal sarcoma in dogs spread as unicellular mammalian organisms for several millennia, once originated from a single cancer cell [89-96]. Thus accumulation of damage is associated with cellular immortality.

### Damage is not sufficient to cause cancer

**Figure 1:**
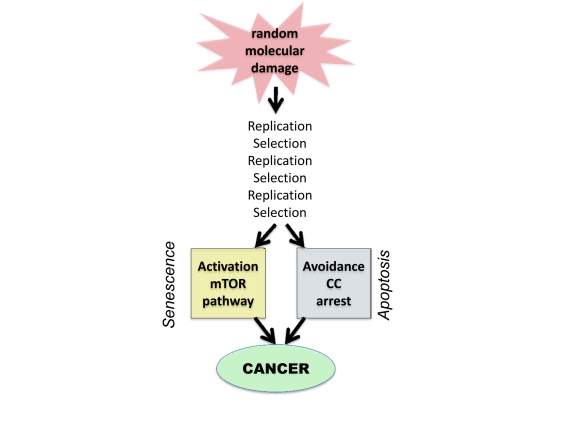
From random damage to cancer Random damage undergoes multiple rounds of replication and selection. Aging is one of selection forces that favors cells with oncogenic mutations. Cancer cell is characterized by (a) activation of growth-promoting pathways such as mTOR and (b) loss of cell cycle (CC) control. Isolated activation of mTOR favors senescence, whereas isolated cell cycle progression may trigger apoptosis.

However, molecular damage is not sufficient either to cause cancer or to hurt organism. This damage is multiplied billions of times via cell replication. Also, cells with random mutations undergo non-random selection (Figure 1).

### Multiplication and selection

A 1 cm tumor contains 10^9^ (1 billion) cells. Therefore, damage does not passively accumulate but is actively multiplied. Cells undergo clonal selection, analogous to Darwinian selection [70, 97-100]. Importantly, most mutations are so called “passenger” mutations that remain random and useless [72, 79, 80, 101]. But nevertheless they do not decrease cell vitality.

### Selective microenvironment

Oncogenic mutations occur randomly. Cancer arises when cellular microenvironment favors oncogenic mutations, creating selective advantage to cells bearing oncogenic mutations. For example, carcinogens not only damage DNA but also cytostatic to normal cells, thus favoring selection of oncogenic mutations that render cells resistant to cytostatic/toxic carcinogens [102, 103]. This is especially apparent with non-damaging carcinogens such as phorbol esters [104]. Cancer therapy can select for additional oncogenic mutations (such as loss of p53), rendering cancer cells not only drug resistant but also increasingly oncogenic [102, 103, 105-108]. Inflammation and chronic infections also favor cancer [109-121]. And the aging microenvironment favors cancer [122-128].

### Aging as selective force

Organismal aging is the most important risk factor in common cancers such as prostate, breast, colon, gastric, lung, pancreatic, skin, brain, thyroid (and so on) cancers as well as melanomas and certain leukemias. Calorie restriction [129-137] and rapamycin [138-141], which decelerate aging, also postpone cancer. Why does aging favors cancer? One explanation is that aging stromal cells secrete factors that promote growth of pre-cancer cells [122, 123, 142-144] and aging is associated with pro-inflammation that favors cancer growth [145-147]. The pro-inflammatory NF-kB pathway is involved in both DNA damage response (DDR), cancer and aging [60, 147-156].

One additional explanation is that chronic overactivation of mTOR renders normal cells irresponsive to growth factors [157]. (In fact, mTOR/S6K renders cells resistant to insulin and growth factors [158, 159]). Then, cancer cells, which are growth signal- independent, acquire selective advantage. In theory, by restoring responsiveness of normal cells to mitogenic signals, treatment with rapamycin can eliminate selective advantage for cancer cells. It was predicted that rapamycin can restore responsiveness of aging cells [157]. In fact, mTOR may cause exhaustion of the proliferative potential of stem cells and, in some studies, rapamycin improved the responsiveness of aging stem cells and immune cells [160-163]. As an example, activation of mTOR promoted leukemia-initiated cells, while depleting normal hematopoietic stem cell. Rapamycin not only depleted leukaemia-initiating cells but also restored normal stem cell function [160, 164]. Thus decreased proliferative potential of normal cells is associated with selective advantage to cancer cells.

### Non-random activation of the PI3K/mTOR pathway

The PI3K/mTOR pathway is universally involved in cancer [37, 165-180]. It is activated by mutations in PI3K, Ras, Raf, non-receptor and growth factor receptor kinases and autocrine growth factors [165, 177, 181, 182]. Also, inactivation of tumor suppressors such as PTEN, AMPK, TSC2, LKB1, NF1 causes activation of this pathway [160, 169, 183-191]. In addition, the hypertrophic effect is often achieved via activation of downstream mTOR targets, translation factors [178]. Finally, p53, which is lost in cancer, is also a suppressor of the mTOR pathway [192-201]. Therefore, it can suppress conversion of cell cycle arrest to senescence [198-204]. In turn, the GF/PI3K/Akt/mTOR pathway drives cellular mass growth, hypersecrtory phenotype, HIF-1 expression, angiogenic phenotype, high levels of glycolysis and biosyntesis (metabolic switch) and apoptosis avoidance [16-35, 205-208]. In other words, it is involved in most of hallmarks of cancer [38, 88], with a notable exception of loss of cell cycle control. On the other hand, the mTOR pathway is involved in senescent phenotype. Therefore, the second alteration in cancer is deactivation of cell cycle checkpoints. Thus cancer cells can be viewed as cycling senescent cells.

### Avoiding cell cycle arrest

In order to proliferate, cell with TOR-activating oncogenes must disable cell cycle control. Inactivation of tumor suppressors such p53, Rb, p16 and activation of c-myc, cyclins D and E, all disable cell cycle control, allowing “pro-senescent” cancer cell to proliferate [209-216]. Still, acute DNA damage, anticancer drugs and induction of p21 or p16 cause cell cycle arrest. Arrested cancer cells rapidly become senescent (geroconversion), revealing their pro-senescent phenotype.

### Oncogenic transformation and gerogenic conversion

There are non-mutually exclusive ways to depict oncogenic transformation, as complementary activation/disabling of signaling pathways [88, 217-225]. Here to compare cancer with aging, I view oncogenic transformation as (a) activation of growth-promoting pathways such as mTOR and (b) loss of cell cycle control. Growth promoting pathways can drive either growth or aging, whereas avoidance of cell cycle arrest precludes aging (Fig. 1). In quiescent cells, activation of growth-promoting pathways (such as mTOR) converts quiescence into senescence, a process named *gerogenic conversion* or *geroconversion* [226, 227]. In proliferating cells, mTOR is fully activated. Induction of cell cycle arrest, without inhibition of mTOR causes gerogenic conversion too. When cell cycle is arrested, growth-promoting pathways drive hypertrophy and aging instead of growth. The difference between quiescence and senescence was recently discussed in detail [227]. Cellular hyper-functions and feedback signal resistance are manifestations of cellular senescence/aging that lead to age-related diseases [227]. These hallmarks result from excessive activation of signaling pathways not from accumulation of damage.

### Why aging is not caused by accumulation of damage

To harbor the active mTOR pathway, cancer cells undergo multiple rounds of selection. In other words, numerous random mutations are selected for non-random activation of mTOR. In contrast it is resting non-dividing cells such as liver, muscle, fat, connective tissue, neurons that undergo aging (geroconversion) in the organism. Not only levels of molecular damage are low in normal cells, but also there is no amplification and selection. So random damage hardly can cause non-random activation of mTOR. Noteworthy, calorie restriction (CR) inhibits mTOR. Even short-term CR suppresses cellular senescence in the organism [228, 229].

### Extragenetic activation of mTOR in aging

mTOR pathway is activated by growth factors, hormones, mitogens, pro-inflammatory cytokines and other secretory molecules and nutrients. Cells can overactivate each other, via positive feedback loops. For example in the liver and fat, hyper-active mTOR causes insulin-resistance, which in turn leads to activation mTOR in beta-cells, which produce insulin. Insulin further activates mTOR in the liver and fat.

### DNA damage response (DDR) and aging

In proliferating cells, mTOR is fully activated. Acute DNA damage induces DDR and cell cycle arrest. If mTOR is still active, such cells undergo geroconversion. Rapamycin and other inhibitors of the mTOR pathway decelerate geroconversion [198, 200, 206, 226, 230-236]). This is how accelerated senescence is usually induced in proliferating cells (in cell culture). However, in quiescent cells with inactive mTOR, DNA damage does not induce sensecence, whereas activation of mTOR does [226, 237].

In oncogene-induced senescence (OIS), DDR causes cell cycle arrest, leading to senescence [238-245]. Noteworthy, most oncogenes that induce senescence (Ras, Raf, MEK, Akt and so on) activate the mTOR pathway. We can call them TOR-activating oncogenes or gerogenes [14], because they are involved in aging from cells to organisms [14, 246, 247]. Loss of PTEN also activates the mTOR pathway, causing senescence [243]. In OIS, oncogenes induce cell cycle arrest but not necessary DNA damage or even DDR [248, 243, 249]. Furthermore, atypical DDR can occur without DNA damage (pseudo-DDR) [231, 236, 250-256]. DDR pathways and the mTOR pathway are interconnected [257-260]. And it seems that pseudo-DDR and DDR are markers of cellular hyper-activation associated with senescence [145] and can be blocked by rapamycin [231].

### Cancer prevention and therapy

Prevention of DNA damage can decrease cancer incidence. For example, non-smoking prevents smoking-induced cancer. Also, cancer can be prevented by decelerating the aging process by calorie restriction and rapamycin. Both calorie restriction and rapamycin delay cancer. Although rapalogs can directly affect cancer cells, rapalogs are only modestly effective as anti-cancer therapy [168, 261, 262], compared with their dramatic preventive effects. In any case, cancer can be prevented without decreasing levels of molecular damage. Furthermore, DNA damaging drugs are cornerstone of cancer therapy. And these drugs are also carcinogens, because anti-cancer and carcinogenic effects are two sides of the same coin [103].

## CONCLUSION

Although molecular damage is typically necessary for cancer initiation, this damage limits life span not because of cellular decline but because of cellular robustness. Damage undergoes multiplication and selection. Aging by itself is a selective force that favors cancer probably because aging cells are signal resistant, thus providing selective advantage to cells that by-pass the need in mitogenic signals. In addition to non-random selection for oncogenic mutations, cancer cells accumulate even higher levels of random “passenger” mutations. Despite that cancer cells are robust. It must be expected that a lower rate of DNA damage in normal cells cannot cause cellular decline. Yes, molecular damage accumulates but is not a driving force for aging. Aging would occur in the absence of any molecular damage. On the other hand, yes, molecular damage is involved in something like cancer that can limit lifespan in mammals to some extend. Noteworthy, worms and flies do not die from cancer. Still they undergo PI3K/TOR-dependent aging [263-269].

As already discussed, if quasi-programmed TOR-driven aging would be eliminated, thus extending lifespan, then accumulation of molecular damage would become life-limiting [10]. In any case, in mammals, cellular aging (characterized by cellular overactivation, hyperfunction and secondary signal resistance) can cause diseases, which lead to organ damage. And cancer, an age-related disease, is not an exception: it kills not because cancer cells fail due to decline but because these cells damage organs. Perhaps, cancer is not the only one damage-related disease among aging-dependent conditions. But a subtle interference of molecular damage with TOR-driven aging will be a topic for another article, which will discuss the intricate relationship between non-random organ damage and random molecular damage.
